# Exogenous myristate promotes the colonization of arbuscular mycorrhizal fungi in tomato

**DOI:** 10.3389/fpls.2023.1250684

**Published:** 2023-11-02

**Authors:** Xiaodi Liu, Zengwei Feng, Wei Zhang, Qing Yao, Honghui Zhu

**Affiliations:** ^1^ Key Laboratory of Agricultural Microbiomics and Precision Application (MARA), Guangdong Provincial Key Laboratory of Microbial Culture Collection and Application, Key Laboratory of Agricultural Microbiome (MARA), State Key Laboratory of Applied Microbiology Southern China, Institute of Microbiology, Guangdong Academy of Sciences, Guangzhou, China; ^2^ College of Horticulture, Guangdong Province Key Laboratory of Microbial Signals and Disease Control, Guangdong Engineering Research Center for Litchi, South China Agricultural University, Guangzhou, China

**Keywords:** arbuscular mycorrhizal fungi, myristate, germ tubes, hyphae growth, colonization, development

## Abstract

Arbuscular mycorrhizal fungi (AMF) can establish symbiotic associations with the roots of most terrestrial plants, thereby improving the tolerance of the host plants to biotic and abiotic stresses. Although AMF cannot synthesize lipids *de novo*, they can obtain lipids from the root cells for their growth and development. A recent study reveals that AMF can directly take up myristate (C14:0 lipid) from the environment and produce a large amount of hyphae in asymbiotic status; however, the effect of environmental lipids on AM symbiosis is still unclear. In this study, we inoculated tomato (*Solanum lycopersicum*) with AMF in an *in vitro* dual culture system and a sand culture system, and then applied exogenous myristate to the substrate, in order to explore the effect of exogenous lipids on the mycorrhizal colonization of AMF. We investigated the hyphae growth, development, and colonization of AMF, and examined the gene expression involved in phosphate transport, lipid biosynthesis, and transport. Results indicate that exogenous lipids significantly stimulated the growth and branching of hyphae, and significantly increased the number of hyphopodia and mycorrhizal colonization of AMF, with arbuscular abundance and intraradical spores or vesicles being the most promoted. In contrast, exogenous myristate decreased the growth range and host tropism of the germ tubes, and largely inhibited the exchange of nutrition between symbionts. As a result, exogenous myristate did not affect the plant growth. This study suggests that lipids promote mycorrhizal colonization by enhancing the growth and development of AMF hyphae and increasing their contact opportunities with plant roots. To the best of our knowledge, this is the first report that shows that lipids promote the colonization of AMF. Our study highlights the importance of better understanding the roles of environmental lipids in the establishment and maintenance of AM symbiosis and, thus, in agricultural production.

## Introduction

Arbuscular mycorrhizal fungi (AMF) can establish widespread mutualistic symbiotic associations with more than 80% of terrestrial plants (Smith and Read, 2010; Tawaraya, 2022; Thismia, 2022). Upon the establishment of symbiosis, they provide mineral nutrients, particularly phosphorus (P) and nitrogen (N), to the host plants (Kobae, 2019; Das et al., 2022; Rui et al., 2022; Yadav et al., 2022). In return, AMF receive carbohydrates and lipids from the host plants (Jiang et al., 2017; Luginbuehl et al., 2017; Sugiura et al., 2020; Wilkes, 2021; Kameoka and Gutjahr, 2022). AMF can enhance the resistance of host plants to various biotic and abiotic stresses, such as nutrient infertility, drought, heavy metal poisoning, low pH, pests and diseases, thereby improving the growth, yields, and quality of crops (Begum et al., 2019; Feng et al., 2020a; El-Sawah et al., 2021; Feng et al., 2022; Ma et al., 2022; Weng et al., 2022; Zhu et al., 2022). In this scenario, AMF are considered to be the most promising “biological fertilizers” and “biological pesticides” (Wilkes, 2021; Anand et al., 2022; Weng et al., 2022), and are likely to play an influential role in the agroforestry systems.

AMF are obligate biotrophs, unable to complete their life cycle or produce daughter spores in the absence of the host plants. AMF lack the cytoplasmic multidomain fatty acids synthase (fatty acids synthase I, FAS I) that catalyzes the *de novo* lipid synthesis (Wewer et al., 2014), but rely on getting lipids from the host plants to sustain their growth, development, and reproduction (Feng et al., 2020a). Keymer et al. (2017) found that the growth of AMF and the accumulation of 16:1ω5 are inhibited in the root of *Lotus japonicus* lipid biosynthesis gene (*KASI* and *GPAT6*)-defective mutants. Subsequently isotope labeling analysis showed that the lipids in wild-type hosts were transferred to AMF across the borders, but this transfer was not observed in the mutants (Keymer et al., 2017). *RAM2* (*Reduced Arbuscular Mycorrhiza 2*) encodes 3-phosphate acyltransferase and is required by the transfer of lipids from host plants to AMF or pathogenic fungi (Bravo et al., 2017; Jiang et al., 2017; Luginbuehl et al., 2017). *STR1* and *STR2* (*Stunted Arbuscule 1* and *2*) encode ATP-binding cassette transporters localized in periarbuscular membrane and are indispensable genes in arbuscular formation (Gutjahr et al., 2012). Jiang et al. (2017) confirmed that *STR* and *STR2* were responsible for transporting the lipids synthesized by *RAM2* to periarbuscular space for absorption by AMF. Taken together, these studies confirm that host-derived lipids play an essential role in the growth and development of AMF.

Lipids are abundant in soil and are the products of plants, animals, and microorganisms (Liang et al., 2019; Angst et al., 2021; Whalen et al., 2022). The input of plant-derived lipids to soils includes three pathways, i.e., litter fall, root exudates, and rhizodeposition (Upadhayay et al., 2020; Dai et al., 2022). The major lipid components of tobacco root exudates are myristic acid, lauric acid, benzoic acid, palmitic acid, cinnamic acid, stearic acid, benzoic acid, and phenylpropionic acid (Li et al., 2017; Banožić et al., 2021; Guo et al., 2021). Animal-derived lipids are mainly the decomposition products of animal carcasses after death (Durães et al., 2010). The major lipids are palmitic acid (C16:0), stearic acid (C18:0), and myristic acid (C14:0) (Algarra et al., 2010). Microbial-derived lipids are mainly the products of microbial membrane degradation, and soil fungi in the rhizosphere provide a large number of unsaturated lipids with pharmacological properties (Tang et al., 2019; Bustamante-Torres et al., 2021). Despite the ubiquity of lipids in soils, however, their impacts on AMF and mycorrhizal colonization are rarely explored.

Lipids are the essential carbon source and energy material for the growth and development of AMF (Jiang et al., 2017; Keymer et al., 2017; Luginbuehl et al., 2017). According to Sugiura et al. (2020), exogenous myristate was absorbed by *Rhizophagus irregularis* DAOM 197198, and induced extensively branched hyphal structure under nonsymbiotic conditions. Therefore, we proposed the hypotheses that (1) exogenous lipids can promote the colonization of AMF in roots and (2) the increased hyphal branching of germ tubes can contribute to the promoted colonization. In this study, symbiosis was established between tomato and AMF in both *in vitro* dual culture system and sand culture system. Exogenous myristate was applied to the substrate to explore the effects of lipids in the environments on AM symbiosis.

## Materials and methods

### Experimental materials

Tomato (Solanum lycopersicum cv. Xinjinfeng No. 1) was used as host plants to establish symbiosis with AMF *R. irregularis* DAOM 197198 or *R. intraradices* BGC JX04B). Tomato seeds were purchased from the market, and the germination rate was above 90%. Tomato hairy roots were obtained by transformation with *Agrobacterium rhizogenes* ACCC 10060 according to the method by Wang et al. (2017). *Rhizophagus irregularis* DAOM 197198 was commercially obtained from Premier Tech Co., Québec, Canada, and propagated in vitro in symbiosis with the tomato hairy roots. *R. intraradices* BGC JX04B was provided by the Institute of Plant Nutrition and Resource, Beijing Academy of Agro-forestry Science. AMF inoculum used in the sand culture system was propagated with clover (Trifolium repens L.) as the host plants for 4 months in the greenhouse. Potassium myristate was used as effective lipid according to Sugiura et al. (2020). River sand was used as the substrate, and washed with tap water after passing through a 2-mm sieve, autoclaved (121°C for 1 h, twice), and oven-dried before using. Plastic pots (top diameter 7.3 cm, bottom diameter 5.9 cm, height 8.8 cm) were used as the container in the pot experiment. Tomato hairy roots and MSR medium were prepared as described previously (Liu et al., 2019).

### Experimental setup and harvest

#### *In vitro* dual culture system (Experiment 1)

A symbiotic relationship was established with tomato hairy roots and AMF in the dual culture system. MSR medium with 0.25% Phytagel (Sigma-Aldrich) containing 0 mM (-myr) or 5 mM (+myr) potassium myristate was prepared to explore the effects of exogenous lipids on the growth and development of AMF with six biological replicates for each treatment.

**Figure 1:**
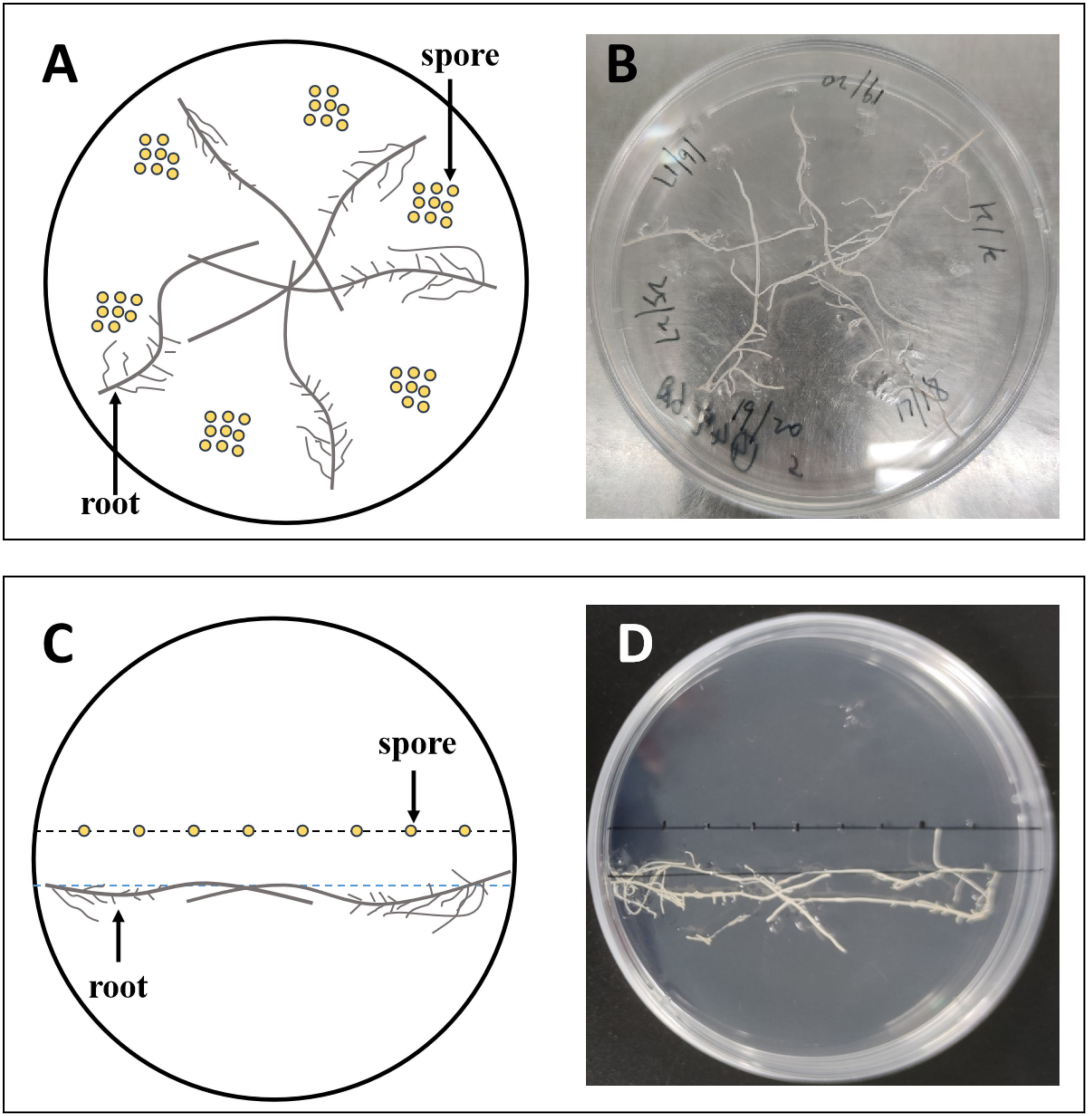
The experimental setup of the dual culture system used in this study. Approximately 50 spores were inoculated on each plate, 8–9 per inoculation site **(A, B)**; 8 spores were inoculated in a row per plate **(C, D)**. **(A, C)** are diagrams of **(B, D)**, respectively.

Fifty mature spores of *R. irregularis* DAOM 197198 were inoculated on the medium. Tomato hairy roots grown on MS medium for 14 days were cut with a lancet and transferred to MSR medium. Five hairy roots of approximately 5 cm length were placed on each plate, and kept approximately 1 cm away from the spores, as shown in **Figures 1A, B**. The plates were incubated in an incubator at 25°C for 16 weeks.

At harvest, the roots in each plate together with the medium were transferred to a 250-mL Erlenmeyer flask containing 50 mL of 10 mM sodium citrate solution (pH 6.0), and incubated for 1 h at room temperature to dissolve the medium on the root and AMF. The roots together with AMF were then transferred with tweezers to the original plate, in which an appropriate amount of distilled water has been added. Extraradical hyphae (EH) and spores were picked out under a stereoscopic microscope, then blotted dry with tissue paper and weighed with a micro analytical balance. The roots were washed with tap water, dried with absorbent paper, and weighed. The roots were then cut into approximately 1-cm root fragments, which were homogenized for measurement of mycorrhizal colonization.

To explore the effects of exogenous lipids on the colonizing pattern of AMF, we inoculated eight spores in a row in each plate, and placed the tomato hairy roots approximately 1 cm away from the spores, as shown in **Figures 1C, D**. There were 20 plates for each treatment, and every 4 plates were combined into one biological replicate at harvest.

#### Sand culture system (Experiment 2)

A symbiotic relationship was established with tomato and AMF in the sand culture system. The experiment was a completely randomized design with a factorial combination of 2 × 2 (2 levels for lipids and 2 levels for AMF inoculation). Two levels of exogenous potassium myristate included 0 mM (−myr) and 5 mM (+myr), and two levels of AMF inoculation included *R. irregularis* (Ri1) and *R. intraradices* (Ri2). Therefore, four treatments were produced, with each containing five biological replicates.

The tomato seeds were surface sterilized with 5% NaClO solution for 10 min, then rinsed with deionized water, placed in a petri dish covered with multi-layer gauze, added with appropriate amount of sterile deionized water, and germinated in an incubator at 27°C in the dark. After germination, the seeds were sown in sterilized perlite and irrigated with deionized water or full-strength Hoagland nutrient solution as necessary. After two sufficiently expanding true leaves emerged, seedlings with similar growth vigor were selected and transplanted into pots. River sand and AMF inoculum (Ri1 or Ri2) were prepared in the pattern of “sandwiches”, namely, 245 g of sand in the bottom layer + 35 g of inoculum in the middle layer + 75 g of sand in the upper layer. One tomato seedling was transplanted into each pot and irrigated with deionized water every day. Hoagland nutrient solution of 1/10 strength phosphorus was supplied weekly with 15 mL per pot each time. For the lipid application, potassium myristate solution of 5 mM was prepared in deionized water and applied to the pots weekly during the first 3 weeks after inoculation with 15 mL each time. The total addition amount of potassium myristate was 0.5 mmol/kg. All pots were placed in a growth chamber (16 h light/8 h dark) at 28°C.

After 6 weeks of growth in the sand culture, the plants were harvested. The shoots were cut off with scissors, and the roots were carefully sampled from the pots and rinsed with tap water before being blotted dry with tissue paper. The fresh weights of the shoots and roots were recorded. The roots were cut into approximately 1-cm root segments, which were homogenized and divided into three aliquots (approximately 0.1 g for each aliquot). Two aliquots were quickly frozen in liquid nitrogen and stored at −80°C for molecular analysis, and the other one was stored at 4°C for the measurement of mycorrhizal colonization (Feng et al., 2020b).

#### Measurement of the number of spores, length of hyphae, number of hyphal branches, growth pattern of germ tubes, hyphopodia, and host tropism (Experiment 1)

The number of spores in each compartment from Experiment 1 was counted under the stereoscopic microscope. Five fields of view were randomly selected on each plate to take pictures, and then the hyphal length was determined using ImageJ (https://imagej.nih.gov/ij/). Twenty spores were randomly selected to count the number of hyphal branches of each spore, including secondary branches, under a stereoscopic microscope.

The percentage of different growth patterns of germ tube from Experiment 1 was evaluated under the stereoscopic microscope. Consistent with Juge et al. (2002), we observed that the germ tubes of different treatments showed two growth patterns: (1) straight germ tubes grew far away from the spores (Type-G), and (2) recurved germ tubes grew around the spores themselves (Type-g). The longer the germ tubes, the greater the ability to infect the roots. Four plates were combined into a biological replicate for statistical analysis. The percentage of germ tubes of different types were measured at 40 days. We counted the number of hyphopodia under the microscope and analyzed the Pearson correlation between mycorrhizal colonization intensity, the number of IH, and the number of hyphopodia with SPSS statistical software.

In this study, we observed that the hairy roots and AMF have the ability to grow toward each other, and called the ability of AMF to grow toward host plant roots as host tropism (Ogawa and Shirasu, 2022a; Sbrana and Giovannetti, 2005; Pineda-Martos et al., 2021; Ogawa et al., 2022b). In addition, some roots grew toward AMF, and the germ tube could contact the roots after spore germination immediately. This type of spore was called “rhizosphere”. The spores with host tropism and growing in the rhizosphere have great opportunity to establish a symbiotic relationship with the roots. The host tropism of the spores from Experiment 1 was evaluated under the stereoscopic microscope 10 days after inoculation (**Supplementary Figure 4**).

#### Measurement of mycorrhizal colonization, the number of intraradical spores or vesicles, and the number of intraradical hyphae (Experiments 1 and 2)

Root fragments from Experiments 1 and 2 were stained with 0.05% trypan blue according to the method of Phillips and Hayman (1970). Briefly, about 100 fine root segments were added to a 5-mL centrifuge tube with 5% KOH solution, and incubated in a water bath at 90°C for 30 min, then rinsed with tap water. Root segments were bleached with 10% alkaline hydrogen peroxide solution for 15 min, acidified in 2% HCl solution for 10 min at room temperature, and then stained with 0.05% trypan blue at 90°C for 30 min. Mycorrhizal colonization was quantified according to the method of Trouvelot et al. (1986) with the software MYCOCALC (https://www2.dijon.inrae.fr/mychintec/Mycocalc-prg/download.html). For each treatment, 30 root segments were randomly selected, mounted onto a slide, and estimated with a microscope (Olympus BX53). F% (the mycorrhizal colonization frequency in the roots), M% (the mycorrhizal colonization intensity in the roots), m% (the relative colonization intensity in colonized roots), A% (the arbuscular abundance in roots), and a% (the relative arbuscular abundance in colonized roots) were calculated with MYCOCALC.

To evaluate the number of intraradical spores or vesicles, we randomly selected 30 root segments of 1 cm length stained with trypan blue, observed, and counted the number of spores or vesicles per centimeter of root segment under the microscope. Thirty mycorrhiza segments were randomly selected to evaluate the number of intraradical hyphae (IH) under the microscope.

### P content

The determination of P content using the molybdenum blue colorimetric method, where KH_2_PO_4_ was used to prepare the standard solution for plotting the standard curve (Thomas et al., 1967).

### RNA extraction and quantitative real-time PCR analysis of selected genes (Experiment 2)

The root samples from Experiment 2 were ground into powder with liquid nitrogen, and then 150 mg of the powder was used to extract total RNA with the RaPure Plant RNA Kit (Magen, Guangzhou) according to the manufacturer’s protocol. The cDNA was synthesized using HiScript II Q RT SuperMix for qPCR Kit (Magen, Guangzhou). To investigate the effect of exogenous lipids on the biomass and functionality (nutrition exchange) of AMF, we analyzed the expression of genes involved in P transport (*SlPT4* and *SlPT5* for phosphate transporter genes identified in tomato) (Parniske, 2008; Harrison et al., 2010), lipid biosynthesis and transport (*RAM2* and *STR2*) (Gutjahr et al., 2012; Bravo et al., 2017), and mycorrhizal colonization-related gene (*R. irregularis elongation factor 1α*, *RiEF1α*) (Pérez-Tienda et al., 2014). The housekeeping gene *Actin* in tomato was used as an internal reference (Feng et al., 2020b; Liu et al., 2020).The primer sequences of all genes were reported previously (Feng et al., 2020b; Liu et al., 2020) and described in **Supplementary Table 1**.

Real-time reverse transcription PCR (qRT-PCR) analysis was performed with a BioRad CFX96 Real-Time PCR Detection System (Bio-Rad Laboratories, Hercules, CA, USA) using PerfectStart™ Green qPCR SuperMix (Transgen, Guangzhou) according to the manufacturer’s protocol. Each 20-μL reaction system contained 10 μL of PerfectStart™ Green qPCR SuperMix (2×), 0.4 μL of forward primers and 0.4 μL of reverse primers, 0.4 μL of passive reference dye, 1 μL of diluted cDNA (1:8), and 7.8 μL of nuclease-free water. The qRT-PCR conditions were as follows: an initial denaturation at 94°C for 30 s, 42 cycles of denaturation at 94°C for 5 s, and annealing at 55°C for 15 s and 72°C for 5 s. The relative expression of target genes was calculated using the 2^−ΔΔCt^ method (Livak and Schmittgen, 2001), with actin serving as an internal standard.

### Statistical analysis

All data are presented as the mean ± standard error of four to six replicates. All treatments and parameters were tested for normal distribution before using other statistical methods. Analysis of variance (ANOVA), two-way ANOVA, Tukey’s honestly significant difference (Tukey’s HSD) test, Independent sample *t*-test, and Pearson correlation were performed with SPSS statistical software (v21.0, SPSS Inc., Chicago, IL, USA).

## Results

### Exogenous myristate promoted the hyphae development of AMF

**Figure 2:**
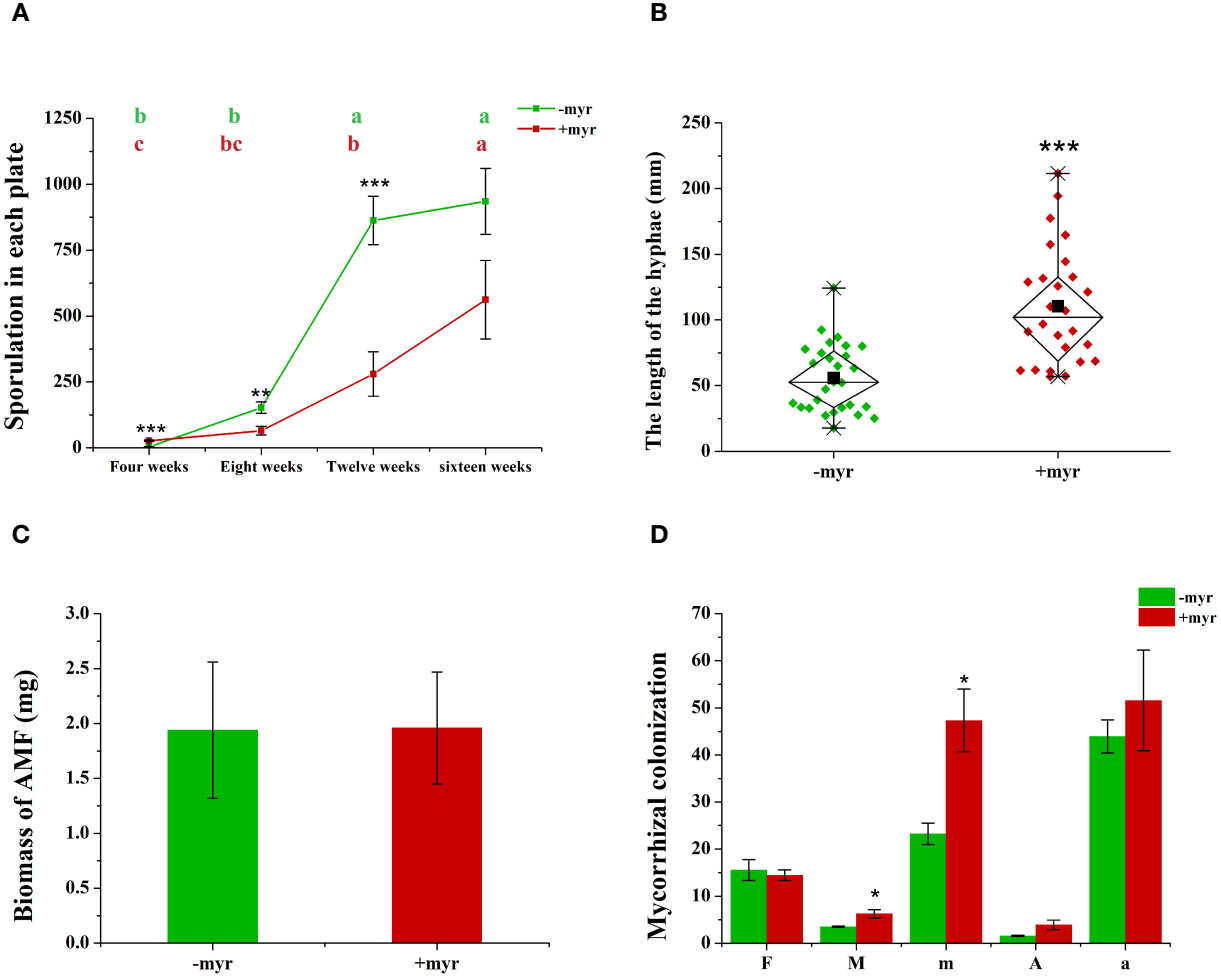
Effect of myristate on the mycorrhizal colonization and development of AMF. **(A)** The sporulation of *R. irregularis* DAOM 197198 at different times (4, 8, 12, and 16 weeks) in each plate. Means (*n* = 4) in each row followed by the same lowercase letter do not differ significantly by the Tukey HSD (*p* < 0.05). Effects of myristate on the hyphae length **(B)**, biomass **(C)**, and mycorrhizal colonization **(D)** of *R. irregularis* DAOM 197198 at 16 weeks. F%: Colonization frequency, M%: Colonization intensity; m%: Relative colonization intensity; A%: Arbuscular abundance; a%: Relative arbuscular abundance. Independent-samples *t*-test (*n* = 4) is performed to evaluate the significance between myristate treatments (*p < 0.05; **p < 0.01; ***p < 0.001); error bars: ± standard error.

At early stages (4 weeks), myristate significantly increased the number of spores, which were directly produced by mother spores without symbiosis with the roots (**Figure 2A**). Subsequently, myristate significantly reduced the number of spores at 8, 12, and 16 weeks (**Figure 2A** and **Supplementary Figure 1**), while in favor of the sporulation at 12–16 weeks (**Figure 2A**), because the spore number increased more rapidly with myristate treatment than without myristate treatment.

**Figure 3:**
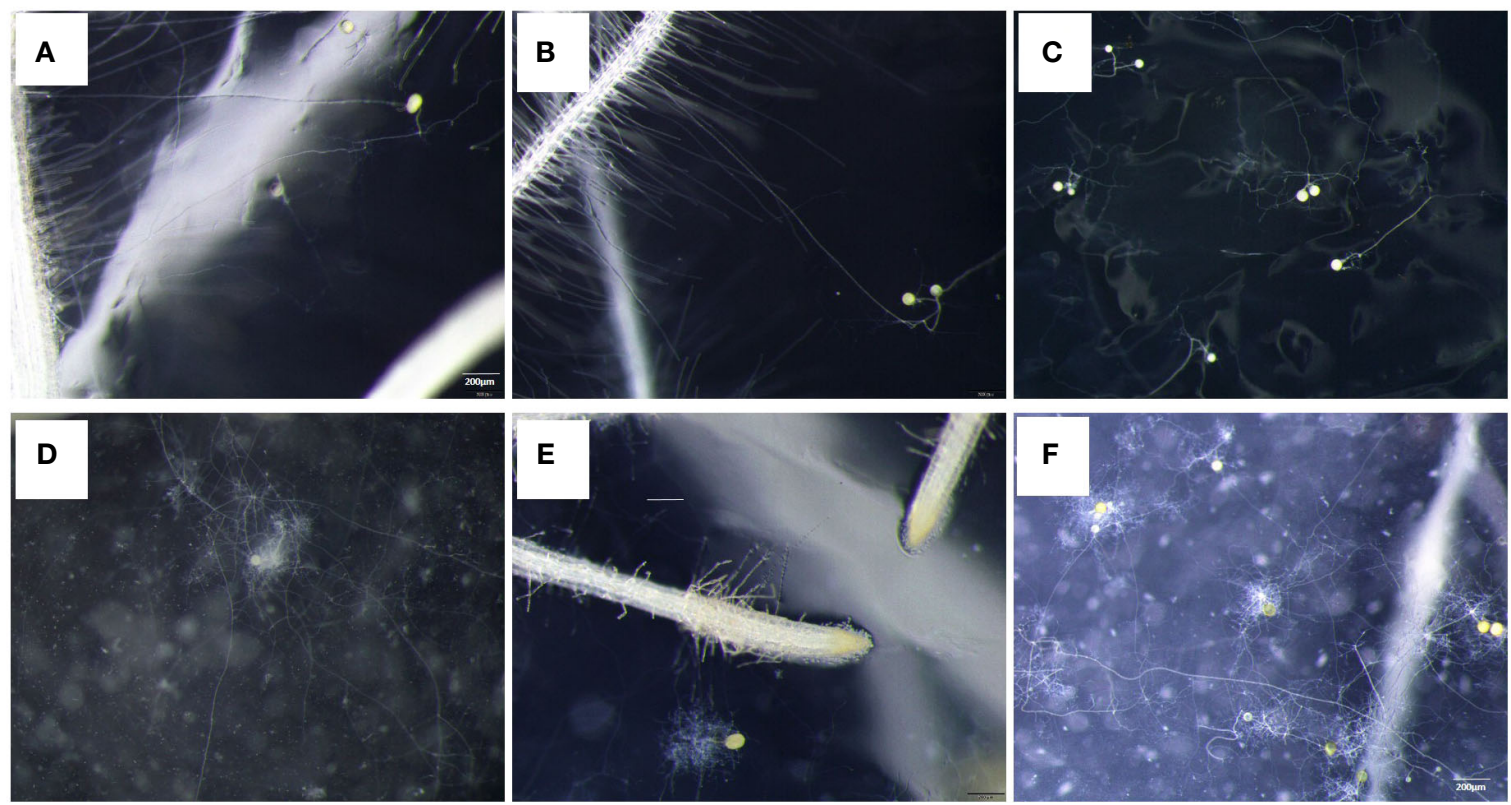
Effect of myristate on the branch of germ tubes of *R. irregularis* DAOM 197198. Each site was inoculated with eight to nine spores, and the tomato hairy roots were placed near the spores. **(A–C)** were treated without myristate; **(D–F)** were treated with myristate. **(A, B, D, E)** show the germination status of individual spores. **(C, F)** were the spore germination status of each inoculation site.

Myristate application significantly promoted the growth of the hyphae. Since each site was inoculated with eight to nine spores (**Figures 1A, B**, **3C, F**), the germ tubes of different spores crossed together during spore germination (**Figures 3C, F**). Without application of myristate, each spore formed only one to two germ tubes, which were less branched (**Figures 3A, B**). With the application of myristate, germ tubes of some spores grow around themselves, form a clump nearby (**Figure 3E**), or part of germ tubes grew farther away with more branched hyphae (**Figure 3D**). We could observe that myristate application promoted the branches of the hyphae and amount of germ tubes, although the hyphal branches formed by individual spore could not be well distinguished (**Figure 3**). Myristate strongly stimulated the EH length of *R. irregularis* DAOM 197198 (**Figure 2B**; **Supplementary Figure 2**), but there was no significant effect on the total biomass of AMF (**Figure 2C**).

In addition, we investigated the mycorrhizal colonization of the AMF. Myristate application had no significant effect on the F% (the colonization frequency), but significantly increased the M% (the colonization intensity) of *R. irregularis* DAOM 197198 (**Figure 2D**). The increase of mycorrhizal colonization suggested that myristate promotes the growth of IH, and/or the increase of hyphopodia caused an increase in the amount of IH. In general, these data demonstrate that the application of exogenous myristate promoted the hyphal development of AMF.

### Exogenous myristate facilitated the hyphopodia

When multiple spores were inoculated on MSR medium at each inoculation site, the state of spore germination and hyphal growth cannot be accurately observed. Therefore, we inoculated one spore per site and eight spores per dish in a row, and just placed one to two tomato hairy roots on the medium to observe spore germination and root infection distinctly (**Figures 1C, D**).

**Figure 4:**
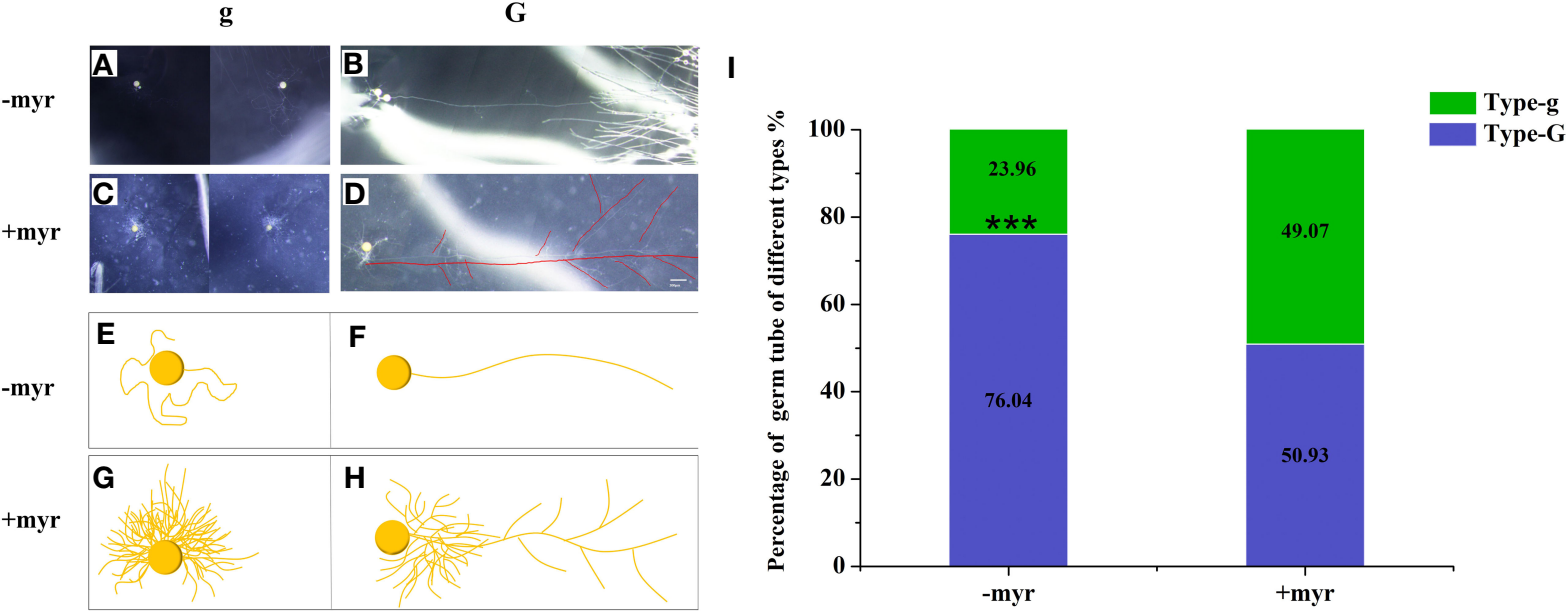
Percentage of different types of germ tubes. Single spore was inoculated near the hairy root of the tomato to observe the growth of the germ tubes. **(A–H)** The morphology of germ tubes in different treatments. **(A, B)** were treated without myristate, **(C, D)** were treated with myristate. **(E–H)** are diagrams of **(A–D)**, respectively. There were two types of germination forms in both treatments, one was that the germ tubes grow only near the spores (Type-g) **(A, C, E, G)**, which has no ability to infect the roots. Another type of germ tubes can grow continuously to a distance (Type-G) **(B, D, F, H)**, which may have the ability to infect the roots. **(I)** Percentage of different types of germ tubes in different treatments at 40 days. “***” in graph **(I)** indicates significant differences among treatments using Independent samples *t*-test (***p < 0.001); error bars: ± standard error.

We observed that the germ tubes of different treatments showed two growth patterns: (1) the Type-g germ tubes grew only near the spores themselves (**Figures 4A, C, E, G**); (2) the Type-G germ tubes can grow continuously to a distance (Type-G) (**Figures 4B, D, F, H**), which may have the ability to infect the roots. The germ tubes of half spores (49.07%) were Type-g, which grew only in a limited area around themselves with application of myristate (**Figure 4I**). By contrast, >75% of the spores showed the G-type germ tubes without application of myristate. Given the above, the growth range of germ tubes was significantly inhibited by myristate application (**Figure 4I**).

**Figure 5:**
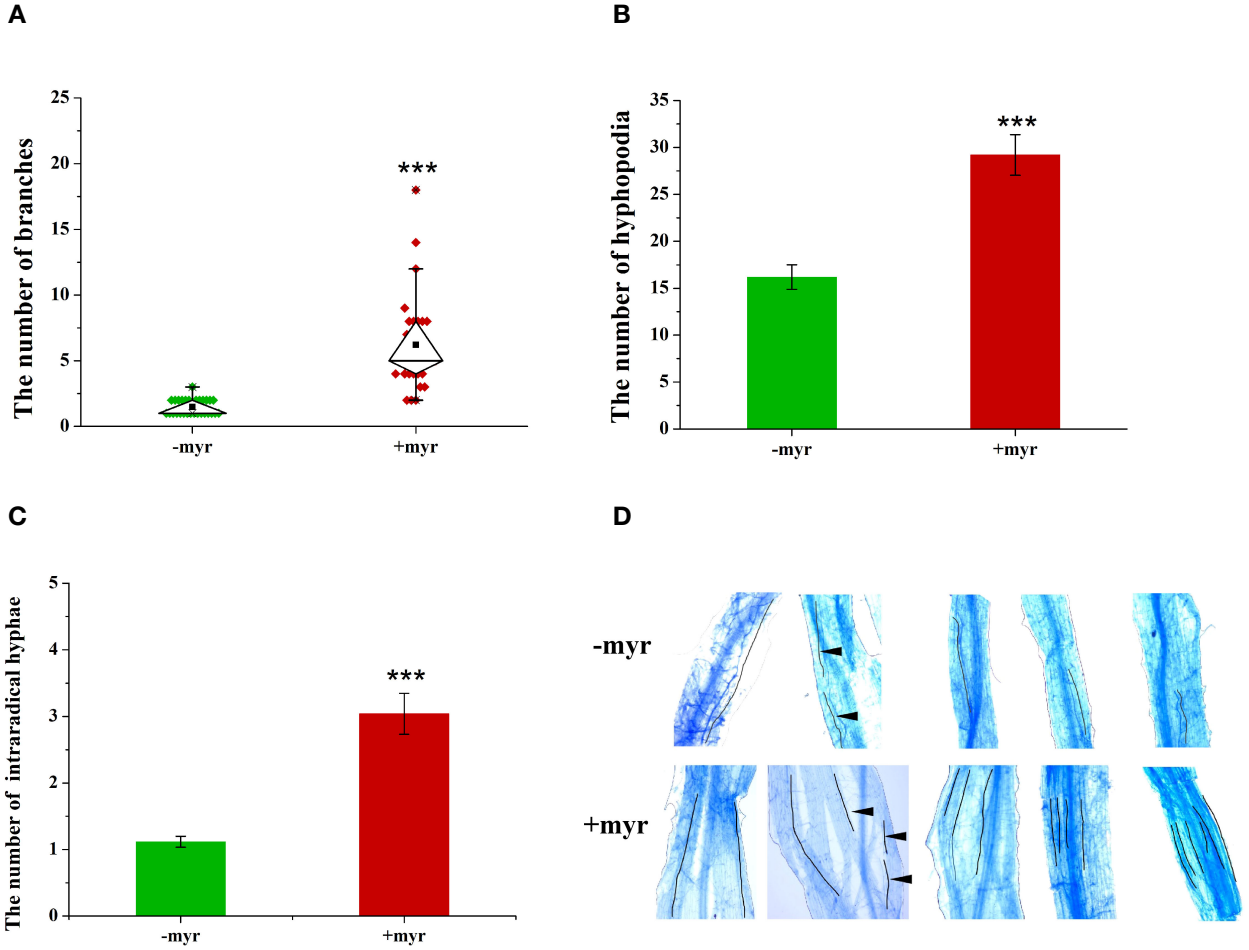
Effect of myristate on the growth and development of *R. irregularis* DAOM 197198. **(A)** The number of germ tubes and branches of each spore; only the branches with hyphal length greater than 0.5 mm were counted, and 20 spores were counted for each treatment at 40 days. **(B)** The number of hyphopodia at 40 days. **(C)** The number of intraradical hyphae. **(D)** Images of IH in different treatments. In −myr, there is usually only one IH in each mycorrhizal segment, whereas in +myr, there are two to six IH in each mycorrhizal segment. The black lines indicate the growth path of IH. The black triangles indicate the disconnection of different segments of the hyphae. “***” above graphs indicate significant differences among treatments using Independent samples *t*-test (***p < 0.001); error bars: ± standard error.

Exogenous application of myristate significantly promoted the branching of germ tubes, and each spore formed approximately 6.2 branches, which was 4.2-fold that of no-myristate application (**Figure 5A**). Meanwhile, exogenous application of myristate significantly promoted the number of hyphopodia, which was 1.8-fold that of no-myristate application (**Figure 5B**). We observed roots stained with Trypan blue and found that discontinuous hyphae or hyphopodia were observed at different locations near a root segment (**Supplementary Figure 3**). The number of IH in each root segment at 4 weeks after inoculation was significantly increased by application of myristate (**Figure 5C**). There was normally only one IH in each root segment without application of myristate, whereas 2-6 IH in each root segment were observed with application of myristate (**Figure 5D**).

We investigated the host tropism of the spores at early stages (10 days) and found that 48.87% of germ tubes with host tropism and 30.07% of spore grew in the rhizosphere without application of exogenous myristate (**Supplementary Figure 4D**). That means germ tubes of approximately 80% spores could infect the roots. In contrast, in the petri dishes containing myristate, approximately 43.08% spores could infect the roots, which was approximately 1/2 that of without myristate (**Supplementary Figure 4D**).

In addition, we analyzed the correlation between mycorrhizal infection intensity, the number of IH, and the number of hyphopodia (**Supplementary Table 2**), and found that the mycorrhizal infection intensity was significantly positively correlated with the number of IH and hyphopodia. The increase in the number of hyphopodia increased the IH, which, in turn, increased the mycorrhizal colonization.

### Exogenous myristate affects the morphology of germ tubes

**Figure 6:**
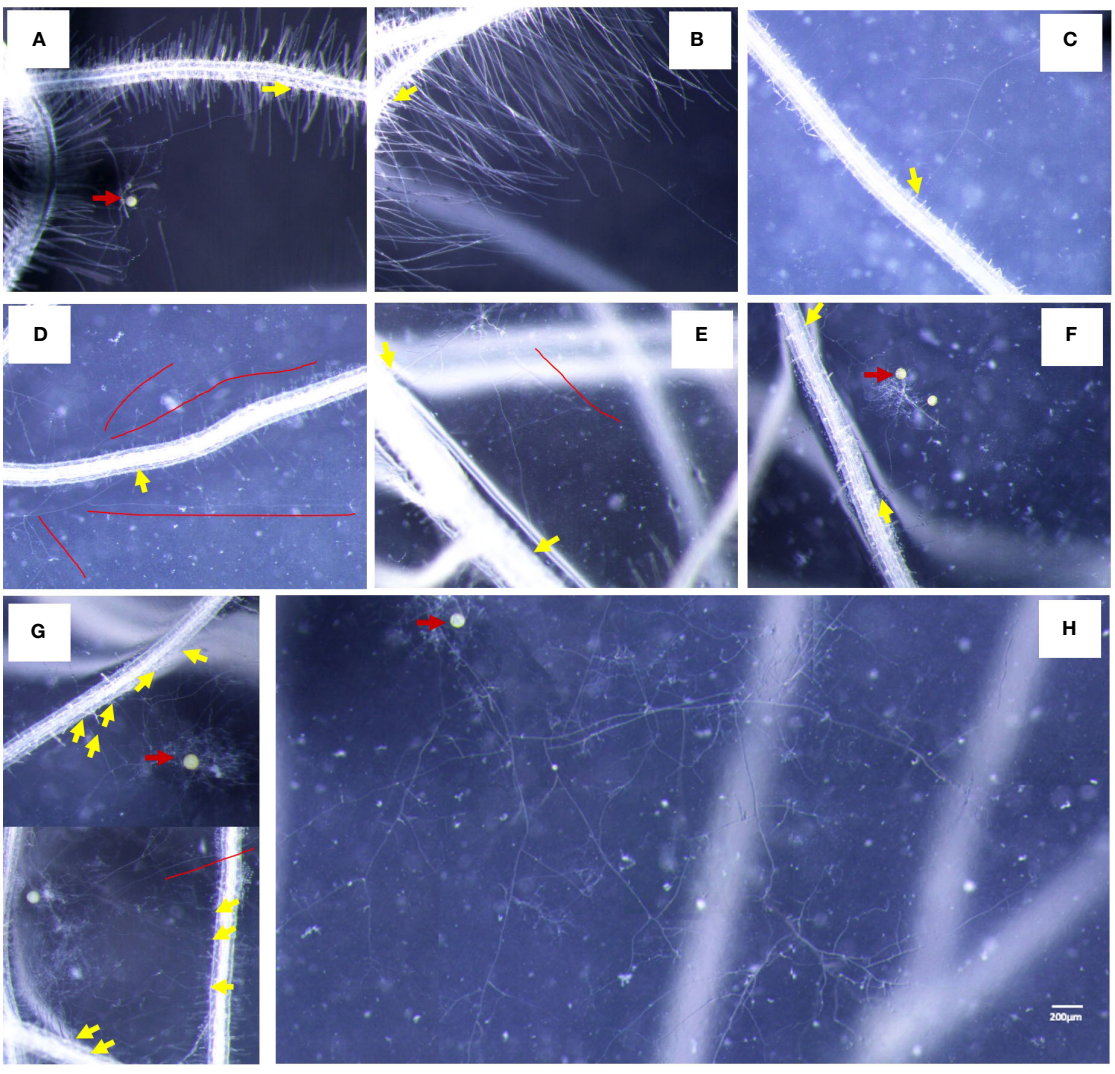
Characteristics of AMF-colonized plants under different treatments. **(A, B)** The colonization status of germ tubes without application of myristate. **(C–H)** The colonization state of germ tubes with application of myristate. **(C, D)** There was one appressorium on the root. **(E, F)** There were two hyphopodia on the root. **(G)** There were multiple hyphopodia on the root. **(H)** Abundantly branched hyphae do not colonize the roots but grow freely. Yellow arrows indicate hyphopodia, red arrows indicate the mother spores, and red lines indicate hyphae that do not colonize the roots.

In the medium without myristate, each spore formed one to two germ tubes (**Figures 3A, B**), and the germ tubes of more than 75% of the spores grew distantly (**Figure 4I**). Each spore formed only one hyphopodium with roots commonly (**Figures 6A, B**).

In the medium containing myristate, all the germ tubes of approximately half spores grew around themselves (**Figures 3E**, **4C, G**). In contrast, for the other half spores, part of their germ tubes grew around themselves, but other germ tubes branched out into the distance (**Figures 3D, F**, **4D, H**). Each spore treated with myristate would form one (**Figures 6C, D**), two (**Figures 6E, F**), or multiple hyphopodia (**Figure 6H**) with the roots, and the hyphopodia in the roots were usually clustered; there were two or more hyphopodia in the vicinity of a root segment (**Figures 6E–G**). Numerous branched hyphae arrived at the roots but did not colonize them; they were growing and branching freely (**Figures 6D, E, G, H**).

### Exogenous myristate promoted AMF colonization

**Figure 7:**
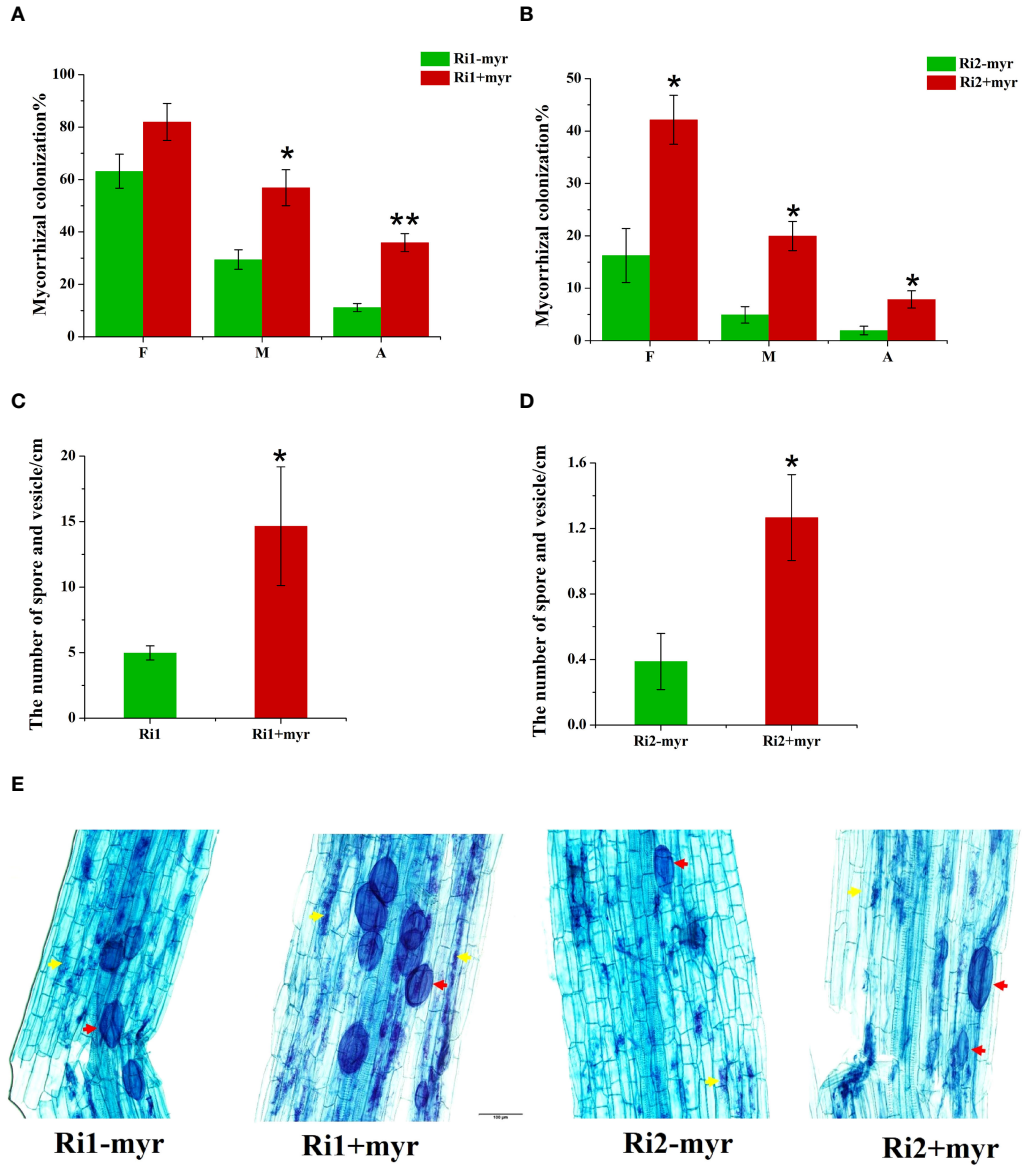
Effect of myristate on mycorrhizal colonization. Mycorrhizal colonization of Ri1 **(A)** and Ri2 **(B)**, F%: Colonization frequency, M%: Colonization intensity, A%: Arbuscular abundance; the number of spore and vesicle of Ri1 **(C)** and Ri2 **(D)**. **(E)** AMF colonization revealed with Trypan blue staining in different treatments. myr: potassium myristate. Independent-samples *t*-test is performed to evaluate the significance between myristate treatments (*p < 0.05; **p < 0.01); error bars: ± standard error.

To clarify the effects of exogenous myristate on AMF colonization, we evaluated the mycorrhizal colonization in the sand culture system based on three parameters, e.g., colonization frequency (F%), colonization intensity (M%), and arbuscular abundance (A%). Our results indicated that the colonization of *R*. *irregularis* (Ri1) was much higher than that of *R*. *intraradices* (Ri2) (**Figures 7A, B, E**). With no application of exogenous myristate, the F%, M%, and A% of *R*. *irregularis* (Ri1) were 63.2%, 29.4%, and 11.1%, in contrast to those of *R*. *intraradices* (Ri2) (16.3%, 4.9%, 1.9%). The results of relative expression level of *RiEF1α* also showed that the mycorrhizal colonization of Ri1 was higher than that of Ri2, and the addition of myristate promoted the colonization in Ri1 (**Supplementary Figure 5**).

Myristate application significantly promoted all three parameters of both AMF species (**Figures 7A, B**; **Table 1**). Specifically, myristate application increased F%, M%, and A% by 29.7%, 93.3%, and 222.4% for *R*. *irregularis* (Ri1) and by 159.3%, 304.5%, and 306.6% for *R*. *intraradices* (Ri2). Meanwhile, the formation of intraradical spores or vesicles was significantly promoted by 177.4% and 194.5% for *R*. *irregularis* (Ri1) and *R*. *intraradices* (Ri2), respectively (**Figures 7C, D**).

**Table 1:** Two-way ANOVA (*p*-value) of the mycorrhizal colonization as influenced by AMF and exogenous myristate.

	F%	M%	A%	Spore
AMF species (AMF)	0.000	0.000	0.000	0.002
Myristate treatment (myr)	0.003	0.000	0.000	0.043
AMF × myr	0.549	0.130	0.001	0.073

F%: Colonization frequency, M%: Colonization intensity, A%: Arbuscular abundance; Spore: the number of spore and vesicle.

### Exogenous myristate inhibited the nutrition exchange

**Figure 8:**
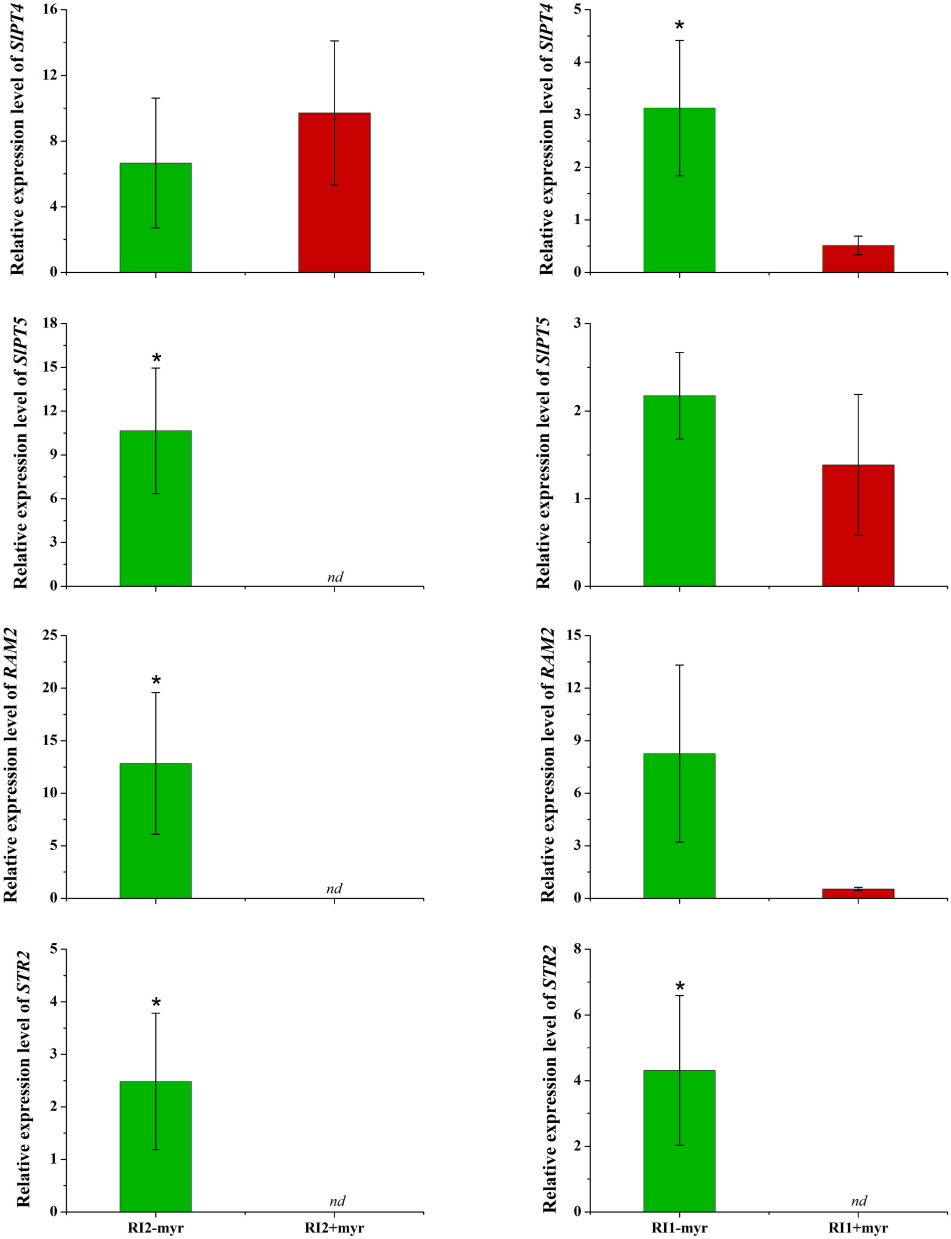
Influence of myristate on the expression of genes encoding plant phosphate transporters, lipid synthesis, and transfer in tomato roots colonized by AMF. Expression of phosphate transfer genes *SlPT*4 and *SlPT*5; expression of *RAM2*, which is responsible for the AMF-specific biosynthesis of lipid in plant endoplasmic reticulum. Expression of *STR2*, which exports β-MAG across the periarbuscular membrane to periarbuscular space. nd: not detected; “*” above graph indicates significant differences among treatments using Independent samples *t*-test (*p < 0.05); error bars: ± standard error.

Root cells provide myristate and take up P from AMF at the symbiosis interface, which are encoded by mycorrhizal-specific lipid biosynthesis and transport (*RAM*2 and *STR*2) and mycorrhizal-specific phosphate transporter genes (*SlPT*4 and *SlPT*5). In the sand culture system, the expression levels of four genes involved in P transport, lipid biosynthesis, and transport in tomato roots were analyzed by qRT-PCR. The results revealed that myristate application had no significant effect on *SlPT*4 and reduced the expression of other genes (**Figure 8**). Specifically, *SlPT*5, *RAM*2, and *STR*2 were completely inhibited in the roots colonized by *R*. *irregularis* (Ri1), and *STR*2 was completely inhibited in the roots colonized by *R*. *intraradices* (Ri2) (**Figure 8**). In both AMF species, the myristate application did not affect the shoot, root biomass, or P content (**Table 2**), although it significantly increased the colonization.

**Table 2:** Influence of myristate on plant biomass and shoot P content of tomato.

AMF species	Treatment	Shoot biomass (g)	Root biomass (g)	Shoot P content (g/kg)
Ri1	−myr	2.24 ± 0.21 a	0.79 ± 0.14 a	0.75 ± 0.02
	+myr	2.23 ± 0.10 ns a	0.79 ± 0.06 ns a	0.73 ± 0.02 ns
Ri2	−myr	1.42 ± 0.05 b	0.58 ± 0.07 a	0.68 ± 0.10
	+myr	1.60 ± 0.08 ns b	0.58 ± 0.05 ns a	0.71 ± 0.03 ns
Two-way ANOVA (*p*-value)				
AMF species (AMF)		0.000	0.046	0.368
Myristate treatment (myr)		0.775	0.999	0.919
AMF × myr		0.437	0.977	0.655

Data are presented as average ± standard error. The “ns” indicates that there is no significant difference in myristate treatment for each AMF (Tukey’s, p ≤ 0.05). All four means in each column followed by the same lowercase letter do not differ significantly by the Tukey HSD (*p* < 0.05).

## Discussion

Lipids are the most abundant component of AMF, mainly including phospholipids (PLs), neutral lipids (NLs) and a small amount of other lipids (Wewer et al., 2014; Campo and San Segundo, 2020; Zhang and Powell, 2021; Kameoka and Gutjahr, 2022). The EH and IH of AMF, especially arbuscules, are characterized by a large membrane surface, which is mainly composed of PLs (Wewer et al., 2014; Campo and San Segundo, 2020; Andrino et al., 2021). Vesicles and spores accumulate a large amount of NLs (triacylglycerol, TAG) (e.g., *Glomus* and *Rhizophagus*) (Gargouri et al., 2021). However, AMF cannot synthesize lipids *de novo*, and need to obtain lipids from roots of host plants for their growth, development, and reproduction. The analysis of multiple AMF genomes reveals that AMF lack FAS I that catalyzes *de novo* fatty acid synthesis (Wewer et al., 2014; Tang et al., 2016; Chen et al., 2018; Kobayashi et al., 2018; Morin et al., 2019). Previous studies have confirmed that the growth and development of AMF mainly rely on obtaining myristate from host plants (Jiang et al., 2017; Keymer et al., 2017; Luginbuehl et al., 2017). In this scenario, we hypothesized that exogenous myristate could increase AMF colonization, which is supported in this study.

Sugiura et al. (2020) investigated the effects of myristic acid and potassium myristate on the growth of AMF. The results showed that there was no significant difference in the standardized growth increment between the two treatments. Previous studies have shown that exogenous potassium inhibited mycorrhizal colonization, and the lowest inhibitory concentration was approximately 40 mg/kg (Ardestani et al., 2011; Kannan et al., 2017; Tshewang et al., 2020). The amount of potassium in the potassium myristate added in our study was 19.5 mg/kg. We assumed that the addition of potassium in this study inhibited mycorrhizal colonization, while our final result was that the potassium myristate significantly promoted mycorrhizal colonization, which further verified that myristate could promote mycorrhizal colonization. Therefore, potassium does not affect our conclusions.

We found that exogenous myristate greatly increased the AMF colonization, especially the arbuscular abundance by two- to threefold in the sand culture system. This result implies that AMF can obtain exogenously applied myristate. Sugiura et al. (2020) confirmed with BODIPY fluorescent staining that the AMF hyphae could directly uptake myristate from the medium. Additionally, they tested eight lipids (C12–C18) and two β-monoacylglycerols, and found that myristate (C14:0) was more effective than others in promoting the growth and sporulation of *R. irregularis* DAOM 197198 under non-symbiotic conditions. In this study, we also found that the increasing magnitude of colonization by exogenous myristate for *R*. *irregularis* (Ri1) (29.7%–222.4%) was lower than that for *R*. *intraradices* (Ri2) (159.3%–306.6%). Considering the much lower colonization of *R*. *intraradices* (Ri2) compared to *R*. *irregularis* (Ri1), it is possible that *R*. *intraradices* (Ri2) obtained less myristate from root cells and thus exhibited a more positive response to exogenous myristate. In addition, exogenous myristate promoted the expression of *RiEF1α*, a gene that represents mycorrhizal infection, in Ri1, but there was no significant difference between the two treatments. This is due to errors between microscopy results and qRT-PCR results (Bodenhausen et al., 2021). In Ri2, *RiEF1α* expression was not detected in either treatment, since the M% was too low to detect the expression of *RiEF1α* (Duret et al., 2022).

In the AM symbiont, AMF spores germinate, then the germ tubes enter root cortical cells and form arbuscules, which represents the interface between AMF and root cells. Previous studies have pointed out that the arbuscular abundance of AMF was closely related to NL accumulation (Feng et al., 2020a; Feng et al., 2020b). Senescent arbuscules contain a large amount of NLs; Kobae et al. (2014) and Feng et al. (2020b) observed that the degradation of arbuscules and accumulation of NLs were synchronized. Kobae et al. (2014) observed the transfer of NLs from IH to EH through real-time imaging technology. In AMF, the spores contain a large amount of NLs, and the NLs in the spores are converted into membrane structures (PLs) in the germ tubes during spore germination (Feng et al., 2020a). The energy and lipids required for germ tube elongation were derived from the spores (Feng et al., 2020a). The PLs in the arbuscules may be hydrolyzed into NLs after collapse, and transferred to AMF spores for storage. Therefore, lipids were extremely important for the germination of AMF and the establishment of a symbiotic relationship with plants and reproduction.

In this study, we observed that exogenous myristate promoted the production of non-symbiotic spore numbers at the initial stage of AMF and reduced the sporulation at 8, 12, and 16 weeks after inoculation, while the gap between the two treatments gradually decreased. Sixteen weeks after inoculation, the addition of myristate significantly increased the sporulation at 12–16 weeks and significantly promoted the EH length of *R. irregularis* DAOM 197198. It is possible that EH preferentially absorbed myristate from the external environment and/or received lipids from collapsing arbuscules for its own growth, and the acquired lipids might subsequently be used to form spores (Kobae et al., 2014; Sugiura et al., 2020; Gargouri et al., 2021; Montero and Paszkowski, 2022). Wang et al. (2017) observed that after *R. irregularis* DAOM 197198 established a symbiotic relationship with tomato hairy roots, large amounts of mycelial networks first formed and then began to form spores. The extensive growth of EH may absorb a large amount of myristate to support the sporulation and also provide sites for sporulation. We infer that the number of spores in the medium that contain myristate would be saturated at the same levels or even higher than that without the application of myristate over a longer incubation period. Both Sugiura et al. (2020) and Tanaka et al. (2022) confirmed that spores formed by absorbing fatty acids from the environment had normal germination and infection ability. Therefore, we reasonably concluded that the spores of AMF formed by exogenous addition of myristate in our study also exhibited normal germination and infection ability. The results indicated no significant difference in germination and mycorrhizal colonization between non-symbiotic and symbiotic spores.

With the application of myristate, half of the spores formed long hyphae, and the hyphae rapidly absorbed myristate from the surrounding environment (Sugiura et al., 2020), forming a large number of highly branched structures. Only a small part of the hyphae went to infect the roots, and most of the hyphae bypassed the roots and grew freely in the medium. The number of branched hyphae and the hyphopodia were both significantly increased by the application of myristate, while the degree of increase at the site of infection (1.8-fold) was smaller than the branches of hyphae (4.2-fold). This suggested that a large number of branched hyphae did not infect the roots in the petri dishes containing myristate.

We could observe one, two, or more hyphopodia at adjacent locations on a root in the medium containing myristate. The number of hyphopodia is only 1.8 times that without myristate. Therefore, there is no significant difference in the colonization frequency, but the colonization intensity of *R. irregularis* DAOM 197198 was significantly increased. This result was observed in both the sand culture system and the *in vitro* culture system. The germ tubes pass through the roots in the form of arbuscules and IH, then extend outside the root to form EH. The addition of myristate significantly increased hyphal branching, EH length, and infection intensity, indicating that myristate plays an important role in hyphal growth and development. The increase of mycelial biomass enhanced the chance of AMF establishing a symbiotic relationship with the roots, which contributed to the increase in mycorrhizal colonization. Because the growth and development of different AMF species in response to lipids are different (Kameoka et al., 2019; Sugiura et al., 2020), the mycorrhizal colonization of different AMF on roots may be different when lipids were present in the environment.

Pearson correlation results shown that the mycorrhizal infection intensity was significantly positively correlated with the number of IH and the number of hyphopodia. The results were easy to understand, and the hyphopodia were the beginning of the symbiotic relationship between AMF and plants. The germ tubes invaded the roots from the hyphopodia, and extended and grew in the roots to form the IH subsequently. Hyphopodia formed the basis of the symbiotic relationship between AMF and plants, and the increase in the number of hyphopodia promoted the increase in the number of IH, which, in turn, promoted the increase in mycorrhizal colonization. Furthermore, elevation of exogenous myristate to IH was higher than that of the number of hyphopodia, which indicated that the number of IH formed by a single hyphopodium increased. The growth of IH is further facilitated by the presence of the exogenous potassium, which was conducive to further increase mycorrhizal colonization.

When AMF establish a symbiotic relationship with host plants, they will obtain sugar and fatty acids from plants to supply their own growth and development. Wu et al. (2019) found that under well-hydrated conditions, inoculation of AMF significantly reduced the content of C14:0 fatty acid in citrus roots compared with non-inoculation treatment. The results suggested that AMF may acquire C14:0 from citrus roots to supply their own growth and development. When C14:0 exists in the environment, AMF can obtain fatty acids from both the environment and the roots of the plant, which may further promote the growth and development of AMF and promote mycorrhizal colonization.

The development of the symbiotic association between AMF and plants resulted from the exchange of signaling molecules between the two partners, which leads to reciprocal benefits (Dhanker et al., 2020). The plant roots secrete strigolactone, which stimulates the AMF hyphal branching and its metabolism (Torabi et al., 2021). AMF releases lipochitooligosaccharides (LOCs) which elicit pre-symbiotic responses in the host root (Li et al., 2022). Plant roots and AMF respond to each other in the course of symbiosis (Dhanker et al., 2020; Nasir et al., 2021), and the roots and AMF are capable of mutual tropism growth. We analyzed the host tropism of AMF (Sbrana and Giovannetti, 2005; Pineda-Martos et al., 2021; Ogawa and Shirasu, 2022a; Ogawa et al., 2022b), and our preliminary results suggest that the host tropism of spores was significantly inhibited by myristate application. The main purpose of this study was to explore the mycorrhizal colonization of AMF, so the hairy roots and spores were not cultured in separate compartments, and we only observed and analyzed the phenomenon of the host tropism in AMF preliminarily. In addition, root growth toward germinating spores was also observed (**Supplementary Figure 4**). We speculated that there was mutual tropism between AMF and hairy roots, which need to be further investigated by designing two- or multi-compartment experiments.

The increased colonization by exogenous myristate did not further promote plant growth. In contrast, the expression of *SlPT5*, *RAM2*, and *STR2* was inhibited by exogenous myristate or was not detectable. Our study showed that the myristate application did not decrease the plant biomass and P content in the inoculated treatments, although the nutrition exchange between AMF and root cells was greatly inhibited. The increase in hyphae biomass by exogenous myristate was accompanied by a large number of hyphae collapse; e.g., the life span of arbuscular is only 7 days (Smith and Read, 2010). AMF biomass (even dead biomass) could promote plant growth (Jansa et al., 2020), and the increase in AMF biomass and mycorrhizal colonization with the addition of exogenous myristate may offset the inhibitory effect. More experiments are needed to investigate the effect of exogenous myristate on AMF function.

## Conclusion

In conclusion, this study demonstrates that exogenous myristate (C14:0 fatty acid) promotes the growth and branch of germ tubes, and increases the number of hyphopodia, the elongation of extraradical hyphae, and the mycorrhizal colonization. These results indicated that exogenous lipids promoted the hyphae growth of AMF and increased the chance of AMF contacting with plant roots, thus promoting the colonization of AMF in tomato roots, including colonization frequency, colonization intensity, and arbuscular abundance. In addition, exogenous fatty acids had different promoting effects on mycorrhizal colonization of different AMF. To the best of our knowledge, this is the first report that exogenous lipids simultaneously increase the colonization but inhibit the nutrient exchange by AMF. Further studies on the effects of exogenous lipids on the function of AMF are needed. In addition, the tropism between AMF and plants may be associated with the root and AMF exudates, which needs to be verified by scientific experiments in the future.

## Data availability statement

The original contributions presented in the study are included in the article/**Supplementary Material**. Further inquiries can be directed to the corresponding authors.

## Author contributions

XL, QY, and HZ designed the study. XL performed the work. XL wrote the manuscript. XL and ZF analyzed the data. XL, ZF, WZ, QY, and HZ revised the manuscript. All authors contributed to the article and approved the final version of this manuscript.
