# Supplementary material for: Exogenous myristate promotes the colonization of arbuscular mycorrhizal fungi in tomato
Source: Front Plant Sci. 2023 Nov 2;14:1250684. doi: 10.3389/fpls.2023.1250684 (PMC10652774; doi:10.3389/fpls.2023.1250684)
Supplement: Supplementary file 1 [file DataSheet_1.pdf]

Supplementary Material

1. Supplementary Tables

Supplementary Table1. Primers for quantitative real-time PCR (qRT-PCR) analysis of genes involved in P transport (*SlPT4* and *SlPT5*), lipid biosynthesis and transport (*RAM2* and *STR2*), mycorrhizal colonization related gene (*RiEF1α*) and the housekeeping gene (*Actin*) in tomato.

Genes	Sequence	Reference
SlPT4	F:GAAGGGGAGCCATTTAATGTGG<br>R:ATCGCGGCTTGTTTAGCATTTCC	Harrison et al., 2010
SlPT5	F:CACTGCCATTATTGAAGGAAATG<br>R:CTAACAAGTCCCATGGTCGG	
RAM2	F: GATGGAACATTACTTCGAGGAC<br>R: CTCCGAGACGAAGTAGTAAAGAAG	
STR2	F: GGACATAGGGAAGCCGATAA<br>R: AGCCCACCCATTGACACTTT	Feng et al., 2020
RiEF1α	F：GCTATTTTGATCATTGCCGCC<br>R：TCATTAAAACGTTCTTCCGACC	
Actin	F:TTCCGTTGCCCAGAGGTCCT<br>R:GGGAGCCAAGGCAGTGATTTC	Chen et al., 2007

Supplementary Table2. Correlation analysis of mycorrhizal colonization, the number of appressoria and the number of internal hyphae (IH).

	M%	m%	Appressoria	IH
M%	1	0.878*	0.890*	0.986**
m%	0.878*	1	0.998**	0.914*
Appressoria	0.890*	0.998**	1	0.920**
IH	0.986**	0.914*	0.920**	1

Note: M%: Colonization intensity; m%: Relative colonization intensity; Appressoria: the number of appressoria; IH: the number of internal hyphae per root segment. These results were analyzed by pearson correlation test (*$P < 0.05$; **$P < 0.01$).

2. Supplementary Figures

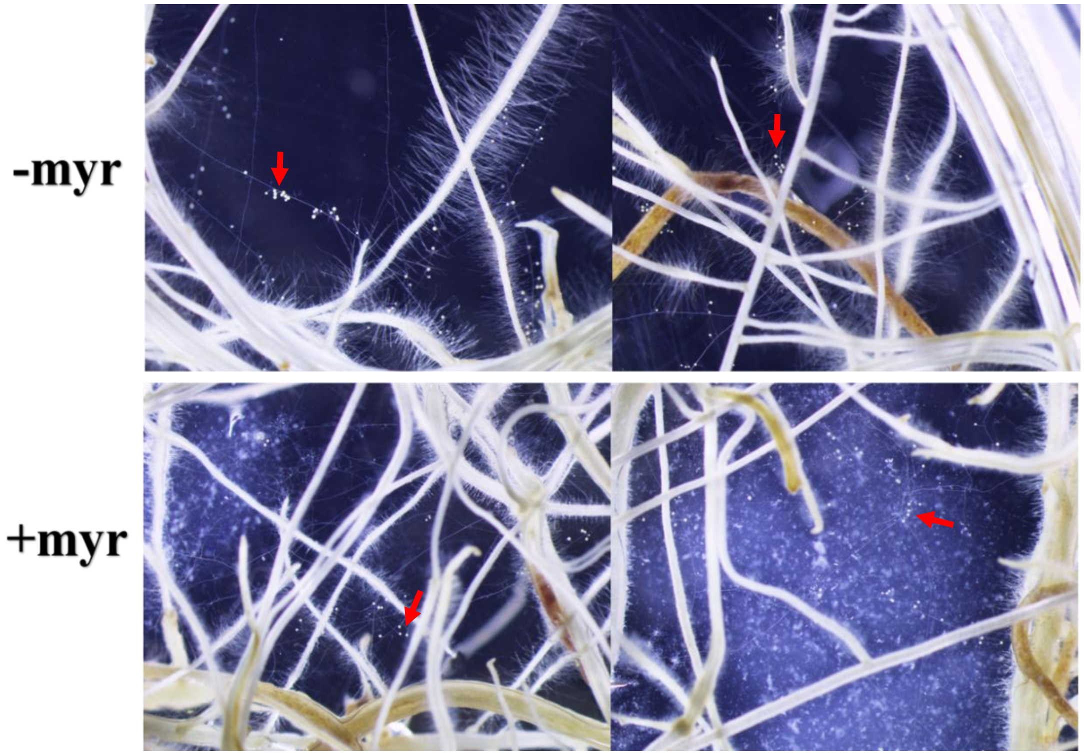

Supplementary Figure 1 Effect of myristate on the number of spore of *R. irregularis* DAOM197198. The red arrows show the spores formed at 16 weeks.

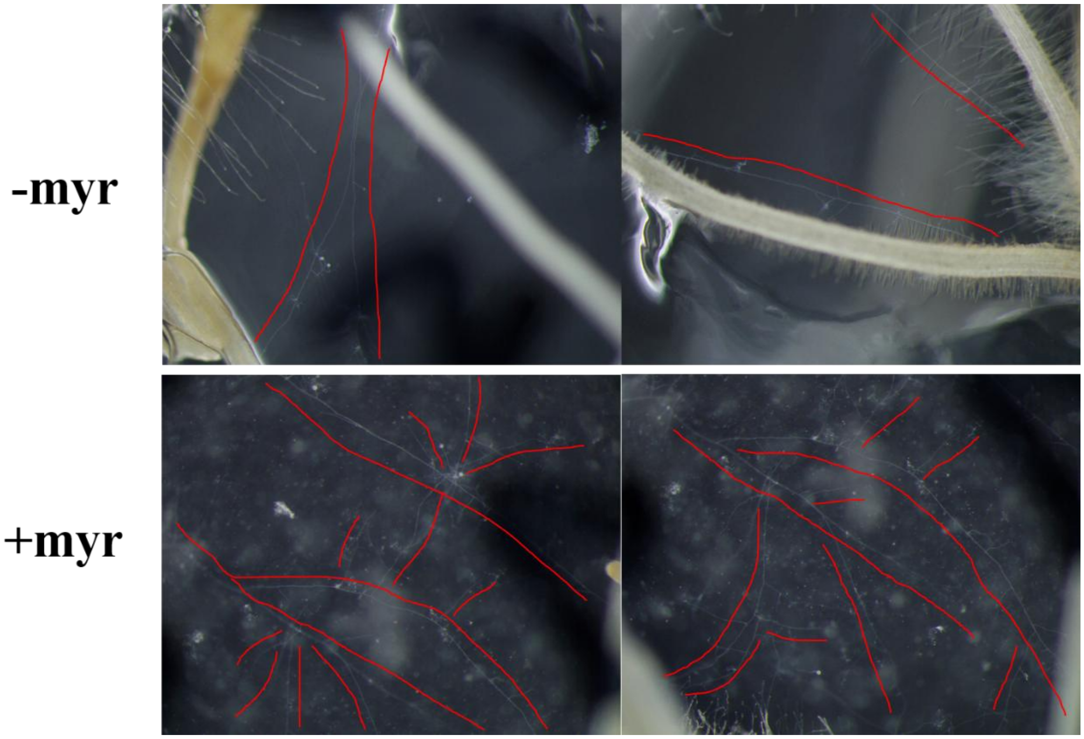

Supplementary Figure 2 Effect of myristate on the hyphae length of *R. irregularis* DAOM197198. The red lines indicate the direction of the extraradical hyphae (EH). Application of exogenous myristate increased the branches and the hyphal length of EH.

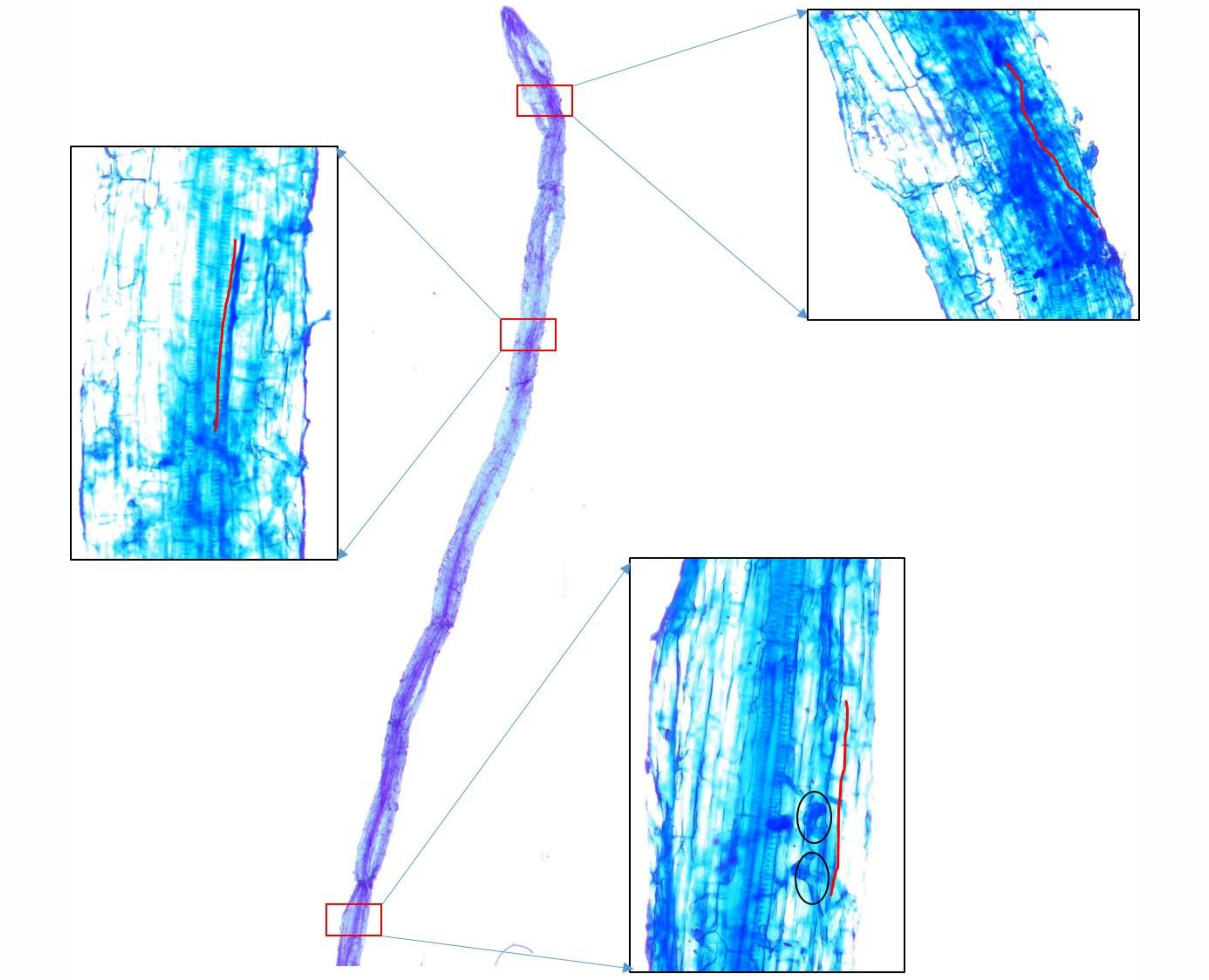

Supplementary Figure 3. In the dual culture system treated with myristate, there were multiple discontinuous IH in mycorrhiza 4 weeks after inoculation. The pictures in the black boxes are the enlarged pictures of the red boxes. The red lines indicate the direction of the intraradical hyphae, and the black circles indicate the arbuscular trunk.

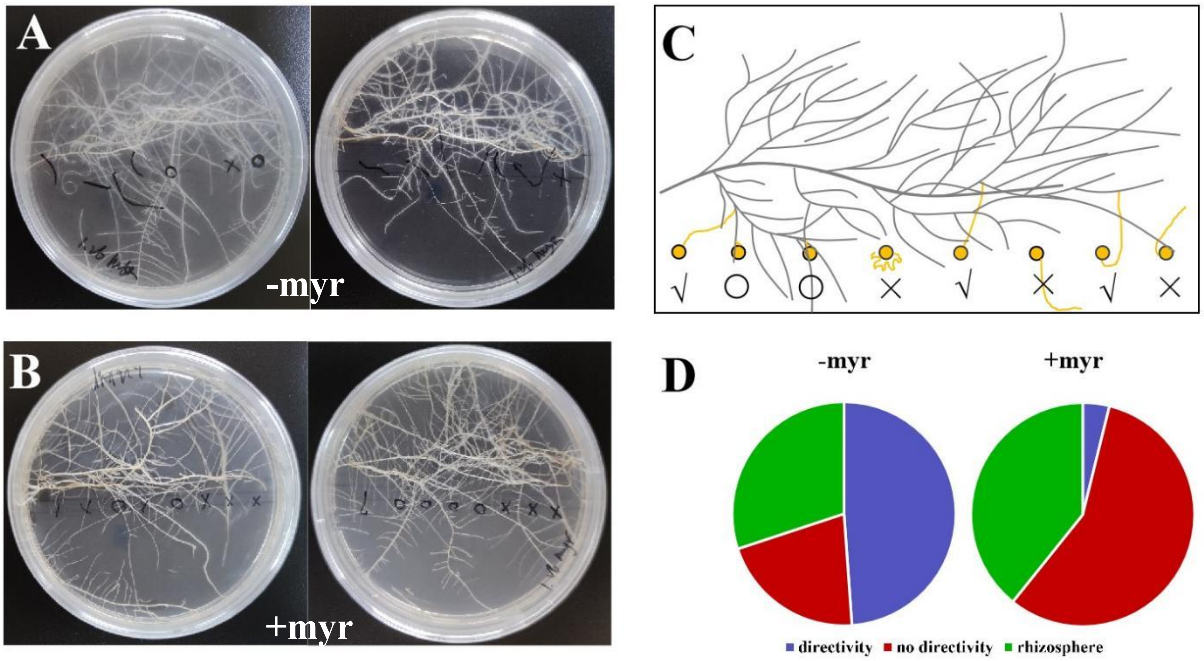

Supplementary Figure 4 Effect of exogenous myristate on host tropism of AMF. Eight spores were inoculated in a line in each plate, and tomato hairy roots were placed about 1 cm away from the spores. We observed that the hairy roots and AMF have the ability to grow towards each other, and called the ability of AMF to grow towards host plant roots as host tropism. Some roots grew toward AMF, and the germ tube could contact the roots after spore germination immediately. This type of spores were called "rhizosphere". **(A)** The plates without myristate; **(B)** The plates containing myristate; **(C)** Diagram of host tropism of AMF; **(D)** Host tropism of AMF spores after germination at 10 days. "√" indicates the spore with host tropism, "×"indicates the spore without host tropism, "○" indicates the spore grow in the rhizosphere.

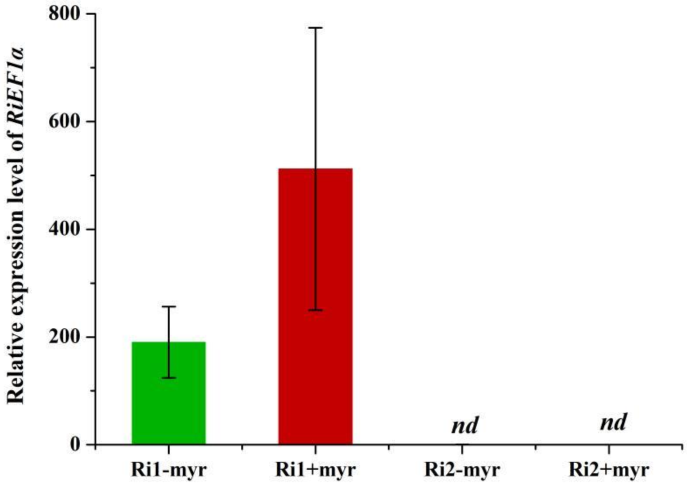

Supplementary Figure 5 Influence of myristate on the expression of *RiEF1α* (*R. irregularis elongation factor 1α*). nd: not detected; error bars: ± standard error.
